# Spontaneous Motor Tempo: Investigating Psychological, Chronobiological, and Demographic Factors in a Large-Scale Online Tapping Experiment

**DOI:** 10.3389/fpsyg.2021.677201

**Published:** 2021-06-22

**Authors:** David Hammerschmidt, Klaus Frieler, Clemens Wöllner

**Affiliations:** ^1^Institute for Systematic Musicology, University of Hamburg, Hamburg, Germany; ^2^Max Planck Institute for Empirical Aesthetics, Frankfurt, Germany

**Keywords:** internal tempo, preferred tempo, slowing with age, time of the day, arousal, inter-tap intervals, circadian rhythm, finger tapping

## Abstract

The spontaneous motor tempo (SMT) describes the pace of regular and repeated movements such as hand clapping or walking. It is typically measured by letting people tap with their index finger at a pace that feels most natural and comfortable to them. A number of factors have been suggested to influence the SMT, such as age, time of the day, arousal, and potentially musical experience. This study aimed at investigating the effects of these factors in a combined and out-of-the-lab context by implementing the finger-tapping paradigm in an online experiment using a self-developed web application. Due to statistical multimodality in the distribution of participants' SMT (*N* = 3,576), showing peaks at modes of around 250 ms, a *Gaussian mixture model* was applied that grouped participants into six clusters, ranging from Very Fast (*M* = 265 ms, *SD* = 74) to Very Slow (*M* = 1,757 ms, *SD* = 166). These SMT clusters differed in terms of age, suggesting that older participants had a slower SMT, and time of the day, showing that the earlier it was, the slower participants' SMT. While arousal did not differ between the SMT clusters, more aroused participants showed faster SMTs across all normalized SMT clusters. Effects of musical experience were inconclusive. With a large international sample, these results provide insights into factors influencing the SMT irrespective of cultural background, which can be seen as a window into human timing processes.

## Introduction

Spontaneous motor tempo (SMT) can be observed in many daily activities such as walking, hand clapping, or swimming. It describes the tempo of self-paced regular and repeated movements and corresponds to the preferred and natural pace to carry out isochronous motor actions, hence SMT is also called internal tempo (Boltz, 1994; Vanneste et al., 2001). The SMT has been described as an estimate of the period of an intrinsic timekeeper, is closely related to the preferred perceived tempo for rhythmic structures such as in music and language (McAuley et al., 2006), and tends to cluster around 500–600 ms (Fraisse, 1982; Collyer et al., 1994; Moelants, 2002). While the exact time-keeping mechanisms remain largely unknown, laboratory research has elucidated some factors explaining the variance between individuals' SMT. It has been shown that the SMT is affected by factors such as age (Baudouin et al., 2004; McAuley et al., 2006; Monier and Droit-Volet, 2018, 2019), arousal (Boltz, 1994; Perilli, 1995), and time of the day (Moussay et al., 2002). Furthermore, it has been reported that musicians have a slower SMT than non-musicians (Drake et al., 2000). In experimental contexts, SMT is typically measured by letting people tap regularly with their index finger of the preferred hand at a pace that feels most comfortable and natural. Recent technology offers solutions to assess the SMT on a large scale in ecologically more valid environments, which allows investigations of naturally occurring conditions such as time of the day. In the current study, we implemented the finger-tapping paradigm in an online study using a self-developed web application, aiming at investigating potential factors for SMT in individuals' familiar environments.

SMT is a central feature in the psychophysics of time perception and plays a crucial role for timing and time processes. According to McAuley and Jones (2003), prevalent models of time experiences can be classified into interval-based and entrainment-based mechanisms. Interval models assume an “internal clock,” which is described in terms of a pacemaker producing periodic pulses (Treisman, 1963; Grondin, 2010; Allman et al., 2014). Entrainment models like the *dynamic attending theory*, on the other hand, propose self-sustaining oscillations as the underlying mechanism of time perception, with attentional pulses reflecting attending energy at a given point of time (Jones and Boltz, 1989; Large and Jones, 1999). Both classes of models (i.e., interval-based and entrainment-based) share the assumption of an intrinsic timekeeper, that is the pacemaker in interval models and the oscillator in entrainment models. SMT can be seen as an estimate of this intrinsic timekeeper, reflecting the pacemaker's preferred pulse rate or the oscillator's preferred period, respectively.

Important aspects that need be taken into account regarding the mechanism for the SMT are anatomical and biomechanical properties of the body (Goodman et al., 2000; Todd et al., 2007), suggesting that the spontaneous pace of cyclic movements may also be influenced by the joints imitating these movements (Peckel et al., 2014; Todd and Lee, 2015). For example, when asked to perform synchronization-continuation motor tasks at rates faster or slower than the SMT, individuals tend to fall back into their SMT over time (Yu et al., 2003; McAuley et al., 2006). The SMT is highly correlated with the preferred perceptual tempo (PPT), which describes optimal processing (i.e., temporal discrimination abilities), suggesting that perceptual and rhythmic motor behaviors share the same underlying mechanism (*preferred period hypothesis*) (McAuley et al., 2006; Michaelis et al., 2014). This assumption is further supported by studies showing a shared resonance frequency at around 2 Hz (500 ms) for the optimal tempo of rhythm perception in music and language (Fraisse, 1982; van Noorden and Moelants, 1999; Moelants, 2002; Ding et al., 2017; Assaneo and Poeppel, 2018), and the execution of predictive (rhythmic) and emergent (cyclic) movements such as finger tapping or walking (Collyer et al., 1994; Goodman et al., 2000; MacDougall and Moore, 2005; Styns et al., 2007; Delevoye-Turrell et al., 2014). Furthermore, the spontaneous pacing of these different body movements have been shown to be similar, as a recent study did not find differences in the SMT between finger tapping, toe tapping, and stepping on the spot, which averaged close to 2 Hz as well, suggesting that the SMT is not influenced by the modality (Rose et al., 2021).

One of the main discussions regarding the SMT concerns factors influencing its pace as a commonly observed result of the SMT is its variability, ranging from 190 to over 1,000 ms (Fraisse, 1982; Collyer et al., 1994; Moelants, 2002; Baudouin et al., 2004). One of the most important findings is a slowing of SMT with age, i.e., older individuals prefer a slower SMT compared to younger individuals (Baudouin et al., 2004). Although slightly varying across different studies, findings suggest an average SMT of 300–450 ms for young children (age 2–7 years) (Provasi and Bobin-Bègue, 2003; McAuley et al., 2006; Monier and Droit-Volet, 2018, 2019), 500–650 for adults (age 18–66 years) and around 1,050–1,125 ms for the elderly (age 66–94 years) (Baudouin et al., 2004; McAuley et al., 2006). These results are consistent with the *slowing-with-age hypothesis* which describes a decline in behavioral speed in the elderly (Surwillo, 1968; Baudouin et al., 2004). This decline of the SMT in the elderly might be caused both by the limits of processing speed (Baudouin et al., 2004) and changes in the neuromuscular system such as reduced muscle strength and endurance. Furthermore, the prefrontal cortex and basal ganglia networks are generally more involved in motor control in the elderly, which are brain regions that are often impaired with higher age (Seidler et al., 2010).

A further factor influencing SMT might be the time of the day. Evidence stems from a study investigating circadian fluctuation of SMT in cycling and finger tapping (Moussay et al., 2002). SMT of finger tapping was measured five times a day and results show that the SMT sped up between 06:00 to 18:00 and slowed down between 18:00 and 22:00, suggesting a direct influence of the circadian rhythm on the SMT. Furthermore, a recent study on cognitive output surrounding sleep investigated the tapping speed of smartphone usage for about three weeks (Huber and Ghosh, 2021). Although not directly comparable, results also show that finger tapping speed on the smartphone (i.e., typing) increased during the morning hours, remained relatively constant during the day and decreased during the night. Thus, these results suggest an influence of the circadian rhythm on the SMT and unconsciously paced finger movements in general, which has been shown to influence cognitive and physiological functions (Valdez and Ramírez, 2012).

Another commonly observed factor modulating the SMT is arousal. The *sympathetic hypothesis* states that higher physiological arousal should speed up the PPT and thus, SMT as well (Holbrook and Anand, 1990). Accordingly, studies found that auditory stimuli inducing varying arousal levels affected the SMT (Boltz, 1994; Perilli, 1995), where high arousal stimuli (i.e., induced short-term stress) led to a faster SMT. In line with this, higher arousal has been shown to be associated with longer time judgments (Burle and Casini, 2001; Ozel et al., 2004; Noulhiane et al., 2007; Wearden, 2008; Grommet et al., 2011; Schwarz et al., 2013), further suggesting that these mechanisms are closely related and that the SMT is linked to an intrinsic timekeeper (Fisher, 2014). Physical activity evokes physiological changes in the body (e.g., heart rate, cortical blood flow) which have been linked to the arousal level (Fisher, 2014; Nobrega et al., 2014). Yet, it remains unclear if changes in heart rate directly affect the SMT. Studies about the relationship between SMT and physical activity remain inconclusive. Whereas one study did find a faster SMT after physical activity (pedaling exercise) (Dosseville et al., 2002), another study did not find a faster SMT after participants performed swimming, running, or wrestling tasks (Sysoeva et al., 2013). The authors explained their null result with the continuous voluntary control with a self-paced speed, yet they noted that further empirical investigations are needed to support this assumption. As mentioned above, induced short-term stress increases the arousal level, causing SMT to speed up as well (Boltz, 1994; Perilli, 1995). Yet, it is unclear if long-term or chronic stress, leading to general physiological changes in the body (e.g., increased heart rate over a longer time period), affects SMT as well (Yaribeygi et al., 2017).

Musical experience has also been reported to influence the SMT, as musicians showed a slower SMT than non-musicians (Drake et al., 2000). Drake et al. found that especially children with musical experience showed a bias toward a slower SMT than children without musical experience. As musical experience has been shown to improve sensorimotor synchronization (SMS) abilities to simple and musical rhythms (e.g., greater synchronization accuracy and rate range), the bias of a faster production rate in non-musicians has been interpreted in such a way that musicians are less restricted in their ability to track auditory-motor events over a longer time span, or in other words, musical experience enables the perceptual organization of events into longer time spans (Scheurich et al., 2018). However, this purely cognitive explanation is somewhat at odds with the assumptions of a low-level biological intrinsic timekeeper, which should not be malleable by a learned cognitive capacity. In sum, it remains unclear why a greater rate range in event tracking may affect the spontaneous and therefore preferred motor tempo.

To sum up, SMT may function as a representation of an underlying intrinsic timekeeper (Large and Jones, 1999; Vanneste et al., 2001; Allman et al., 2014). It slows down with age, potentially due to a decline in processing speed and changes in the neuromuscular system (Baudouin et al., 2004; McAuley et al., 2006; Hunter et al., 2016), it may further be influenced by the circadian rhythm (i.e., time of the day) (Moussay et al., 2002), and is likely to speed up with higher arousal levels (Boltz, 1994). Furthermore, it has been reported that the SMT is generally slower for children with musical experience than children without musical experience (Drake et al., 2000). It is not known if long-term or chronic stress affect SMT, which might be the case as long-term stress leads to general physiological changes in the body (Yaribeygi et al., 2017).

This study aimed at investigating these factors and their effects on the SMT for the first time with a large international sample, and further attempted to close the gap between lab-based studies using the finger-tapping paradigm as a measure of SMT and individuals' familiar environment by implementing this paradigm in an online experiment using a web application. We hypothesized that (i) SMT slows down with age, that (ii) SMT is influenced by the time of the day, and that (iii) arousal and long-term stress speed up the pace of the SMT. Furthermore, we investigated if participants with more musical experience prefer a slower SMT than participants with less musical experience.

## Methods

### Participants

A total of 5,966 participants took part in the study, out of which data from 3,576 participants were used for further analysis due to exclusion criteria (see section “Data Analysis”). The mean age of participants was 27.64 years (*SD* = 7.61, range: 7–49 years) and 64% were male (1% other). Participants were from 74 different countries, yet the majority was from China (81.2%), and 62.1% of them worked or studied for 40 h or more in a typical week. They were relatively inexperienced in terms of music making, *M* = 2.07 (*SD* = 1.39), whereby participants rated their experience in music making on a scale from 1 = “never” to 6 = “I am a professional.” On average, participants needed *M* = 1.59 trials (*SD* =1.01) to meet the criterion for a successful tapping trial (see section “Design and Procedure”). The number of tries needed for a successful tapping trial depended on musical experience, *Spearman's rho* = −0.09, *p* < 0.001.

All participants gave informed consent online in accordance with the Declaration of Helsinki. The procedures were in accordance with the guidelines of the Ethics Committee of the Faculty of Humanities at University of Hamburg.

### Design and Procedure

This study had a between-participant design and was divided in three parts. The first part consisted of demographic information. The second part consisted of the main experiment, in which the SMT was measured. In the third part, variables including arousal level, musical experience, and the long-term stress were collected (see Supplementary Material).

Participants were invited to test how good their “inner timing” is, meaning how even they can tap without external influences such as music, and how they perform compared to others. After providing informed consent, participants first entered their demographic information such as age, gender, country of residence, and the population size of the city/area they currently live in. Furthermore, they stated if they had taken part in the experiment before, as access to the web application was not restricted. Then they were asked to tap steadily for 15 s with their finger on a device of their choice (PC keyboard or mouse, touchscreen of a tablet, or smartphone). The task was “to keep the time between each tap as even as possible” and to “choose a pace that feels most comfortable and natural to you right now.” During the tapping trial, a visual bar was running continuously from left to right indicating for how much longer they needed to tap (see Supplementary Material). After the finger-tapping task, feedback on their tapping consistency was given in terms of the evenness of taps, whereby 100% represent no variability at all between inter-tap intervals. Furthermore, they were informed how well their score was compared to the previous sample of participants. The goal of this feedback was to make the experiment more appealing in order to reach a higher number of participants. They were not informed that the chosen pace of the taps was the main measure for this study. If the tapping variability, measured as the coefficient of variation (CV) of inter-tap intervals, was too high (maximum CV = 0.1) or the total number of taps was less than eight, participants were automatically asked to repeat the tapping task which was not limited in terms of number of tries. This strict criteria for the tapping variability were chosen as it warrants correct task execution, since the recording time of taps was relatively short. If a tapping trial was accepted, participants could proceed to the third part of the experiment. In this part, participants rated their current arousal level, ranging from 1 = “very calm” to 5 = “very excited,” their musical experience by asking if they make music, ranging from 1 = “never” to 6 = “I am a professional,” their average working/studying hours in a typical week, and filled out the short-form *Perceived Stress Scale* (PSS-4), in order to assess long-term stress (Cohen and Williamson, 1988). Further data included the date and local time of test execution and user agents of participants (device brand and model, operating system, and web browser). The experiment was available in four languages (English, German, French, and Mandarin) and participants could choose between them by clicking on the corresponding flag. The default language was English, and translations were carried out by native speakers using the back-translation method.

The browser-based web application was based on the MEN stack, and the frontend was programmed in Javascript using the Node.Js Express Framework. The backend was hosted with NGINX on a Linux server from the University of Hamburg and the used database was MongoDB. The web implementation and programming were done by Simon Mayrshofer and is publicly available on Github (https://github.com/g-mac/slomo).

### Data Analysis

The mean inter-tap intervals (ITI) of each participant were calculated as a measure of their SMT. Before applying statistical analysis, data was filtered according to the following conditions: all responses needed to be given by a single participant (*N* = 3,986), and if they took part in the experiment more than once only the first participation was considered (*N* = 3,704). If the mean ITI of a participant was faster than 100 ms, then their data was not further considered (*N* = 3,703), since 100 ms can be assumed to be the motoric lower limit for finger tapping. Furthermore, an outlier detection using 1.5 interquartile range was applied on age (*N* = 3,577). This factor informed the most about potential incorrect responses given by participants (e.g., one participant claimed to be 1 year old) and the outlier detection offered an objective approach for their exclusion. One participant was removed from the study who claimed to be from Antarctica. The data cleaning approach resulted in a data reduction from *N* = 5,966 to 3,576 (59.9%). The different hard- and software used by the participants (see Supplementary Material) did not influence the SMT results [device types: *t*_(1, 3, 574)_ = −1.40, *p* = 0.16, *d* = −0.5, operating systems: *F*_(4, 3, 571)_ = 0.47, *p* = 0.44, η^2^ = 0.001, browser: *F*_(6, 3, 569)_ = 1.56, *p* = 0.15, η^2^ = 0.003], suggesting that any such potential influence was mitigated which was expected as time point differences were measured so a device effect could only be caused by a varying internal device latency.

**Figure 1:**
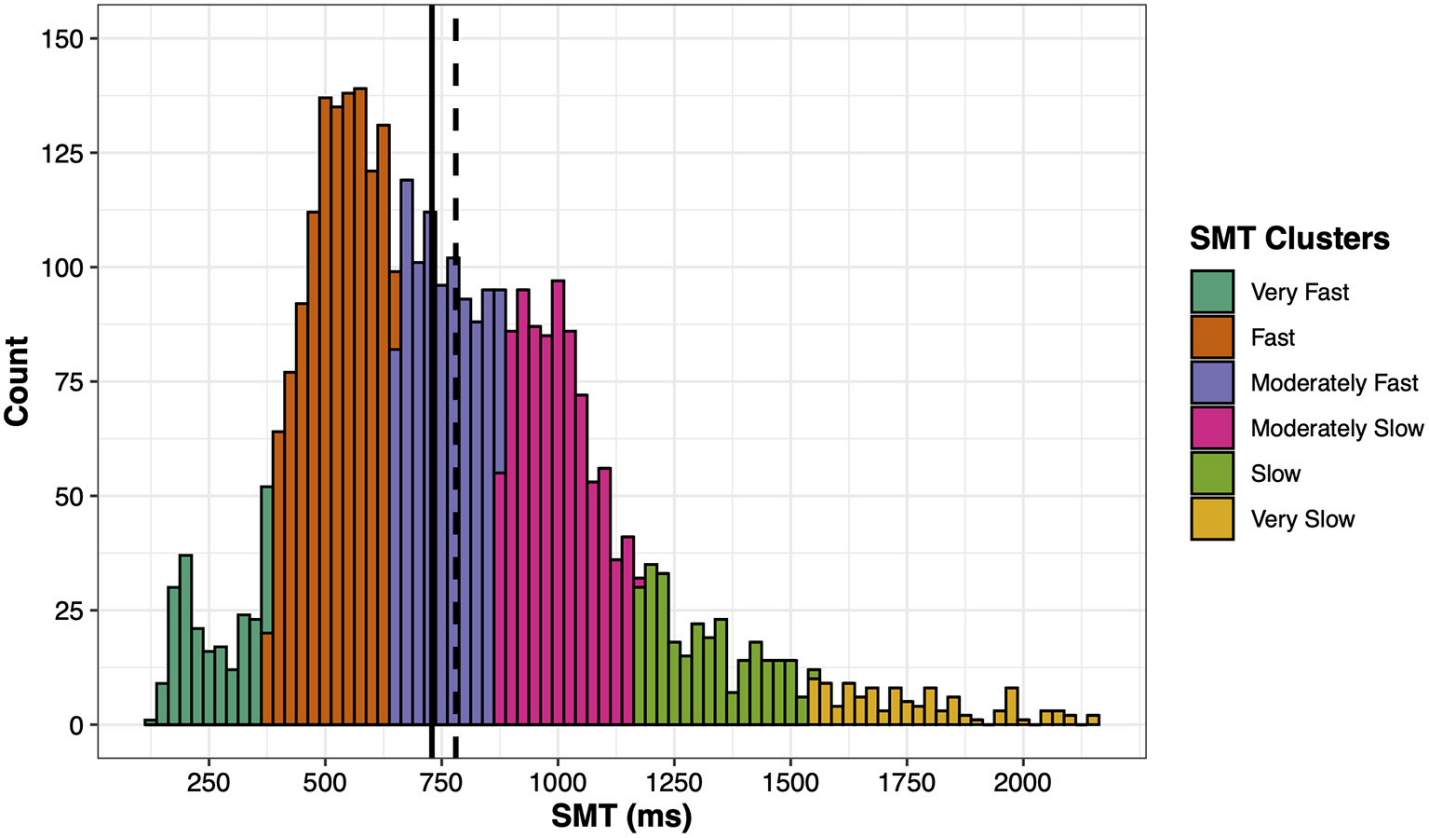
Distribution of the SMT data. The solid line indicates the median and the dashed line the mean. Each color represents a SMT cluster. The bar width represents 25 ms.

Inspecting the distribution of SMT data, a *Shapiro–Wilk* test (*W* = 0.95, *p* < 0.001), *measure of skewness* (0.91, *SE* = 0.04), and *Kurtosis* (1.25, *SE* = 0.08), suggested a left skewed, super Gaussian and leptokurtic distribution (see Figure 1). Furthermore, the data shows multiple modes (peaks) suggesting a multimodal distribution. In order to account for this multimodality by identifying different modes, a *Gaussian mixture model* was applied using the Mclust package in R. A systemic clustering was applied on the SMT and the coefficient of variation (CV) of SMT including 10 different geometric characteristics and up to nine mixture components (i.e., number of clusters). Participants' CV was included in order to stabilize the model and improve the clustering. As we were only interested in the clustering of the ITIs and not their corresponding variability, the final model grouped participants into six different clusters consisting of a spherical distribution with equal volume and equal shape (“EII”). This model identified the fastest cluster peaking around 200–250 ms, which is small but visible (see section “SMT Distribution and Clusters”). Next, separate *Analyses of Variance* (ANOVAs) were applied using these SMT clusters as an independent variable and age, arousal, long-term stress, and musical experience measures as fixed factors. *Post-hoc* comparisons of these ANOVAs were calculated with Tukey adjustments. As time of the day is circular, analyzing differences between the SMT clusters using a conventional ANOVA is not feasible. Thus, the local times of test execution were converted into circular vectors in order to apply a circular ANOVA to compare the mean time of the day between SMT clusters using the Circular package in R. Circular *post-hoc* comparisons were based on the method from Tasdan and Yeniay (2018) and adjusted according to Holm.

In order to check for general predicators of the SMT and to ensure homoscedasticity, ITIs were cluster-wise z-transformed before applying a multiple regression model with the factors age, arousal, musical experience, and long-term stress. The cluster-wise transformation was done in order to account for the different modes of multiples around 250 ms. Data processing and analysis for the whole study was done in R (R Core Team, 2020).

## Results

### SMT Distribution and Clusters

Participants' individual SMT was calculated by taking the mean of their inter-tap intervals (ITIs). Overall, the mean SMT of all participants was 780 ms (*SD* = 328) and the median ITI was 729 ms, ranging from 123 to 2,150 ms. Table 1 shows the descriptive statistics of fixed factors and Table 2 the correlation matrix between them including the SMT. As Figure 1 shows, the distribution of SMTs across all participants is multimodal (see section “Data Analysis”). In order to statistically account for this data distribution, a *Gaussian mixture model* (GMM) was applied before further testing for influences on the SMT. The GMM grouped the participants into six SMT clusters (see color scheme in Figure 1). These clusters differ in terms of participants' ITIs, thus each cluster represents a different tempo range of SMTs. As Table 3 shows, the mean SMTs for each cluster resulted in approximate multiples of 250 ms, which might be indicative of a base frequency of around 4 Hz.

**Table 1:** Descriptive statistics of fixed factors.

**Factor**	**Mean**	**Median**	**Standard deviation**	**Range**	**Minimum**	**Maximum**
Age (years)	27.65	26.00	7.61	42.00	7.00	49.00
Arousal (rating scale)	2.21	2.00	0.95	5.00	1.00	5.00
Long-term stress (PSS-4)	7.60	8.00	2.96	16.00	0.00	16.00
Musical experience (rating scale)	2.10	1.00	1.39	6.00	1.00	6.00

**Table 2:** Correlation matrix using *Spearman's rho*.

	**SMT (ms)**	**Age**	**Arousal**	**Long-term stress**	**Musical experience**
SMT (ms)					
Age	0.12*				
Arousal	−0.06*	−0.10*			
Long-term stress	−0.01	−0.14*	0.15*		
Musical experience	−0.04	−0.18*	0.12*	−0.04	

*Values indicate the correlation coefficient based on N = 3,576. Asterisks indicate significance (adjusted α = 0.005)*.

**Table 3:** Descriptive statistics of each SMT cluster.

**SMT cluster**	**Number of participants**	**Mean ITI (ms)**	**Median ITI (ms)**	**Standard deviation (ms)**	**Range (ms)**	**Minimum (ms)**	**Maximum (ms)**
Very Fast	223	265	261	74	123	123	375
Fast	1,184	525	529	70	267	375	642
Moderately Fast	925	754	750	67	232	642	875
Moderately Slow	852	997	992	77	289	876	1,164
Slow	283	1,314	1,301	106	373	1,167	1,541
Very Slow	109	1,757	1,729	166	607	1,543	2,150

### Differences Between SMT Clusters

In order to check for differences of age, time of day, arousal, long-term stress, and musical experience between the SMT clusters, separate *Analyses of Variance* (ANOVAs) were performed.

#### Age

**Figure 2:**
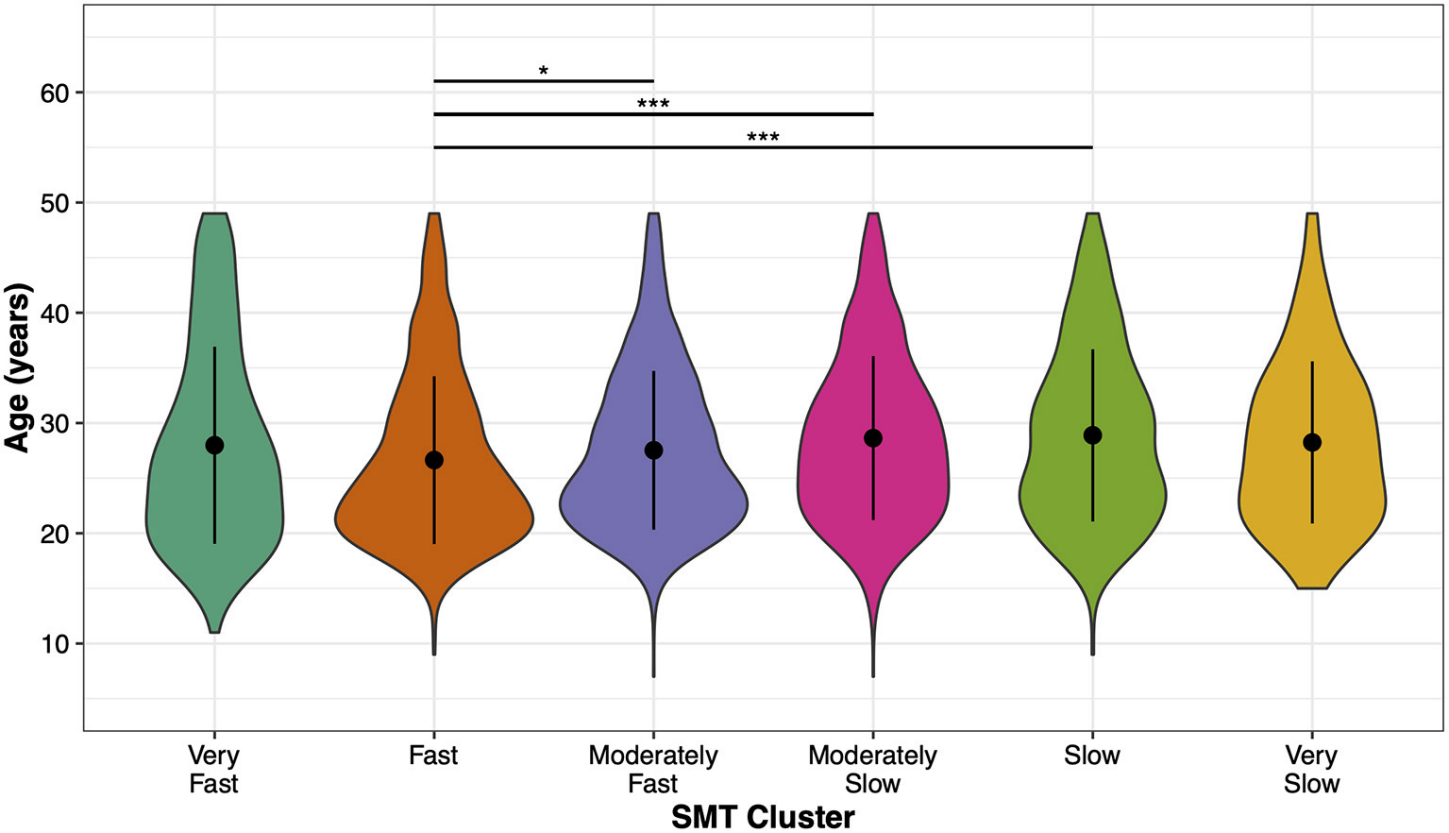
Age distribution between SMT clusters. Mean values (black points) and standard deviations (error bars) of participants' age for each SMT cluster. Colored areas show the age distribution. Asterisks indicate significant differences: *p < 0.05, **p < 0.01, and *** *p* < 0.001.

The ANOVA on Age showed a significant main effect, *F*_(5, 3, 570)_ = 8.79, *p* < 0.001, η^2^ = 0.01, suggesting that the mean age of participants differed between the SMT clusters (Figure 2). *Post-hoc* comparisons show that participants in the Fast SMT cluster were younger than participants in the Moderate Slow (*p* < 0.001) and Slow SMT clusters (*p* < 0.001). Furthermore, Age differed between the Moderately Fast and Moderately Slow SMT clusters (*p* = 0.04), showing that the participants in the Moderately Fast SMT cluster were younger. All other comparisons were non-significant (all *p* > 0.05). These results indicate with increasing age, participants were more likely to be classified in a slower SMT cluster.

#### Time of the Day

**Figure 3:**
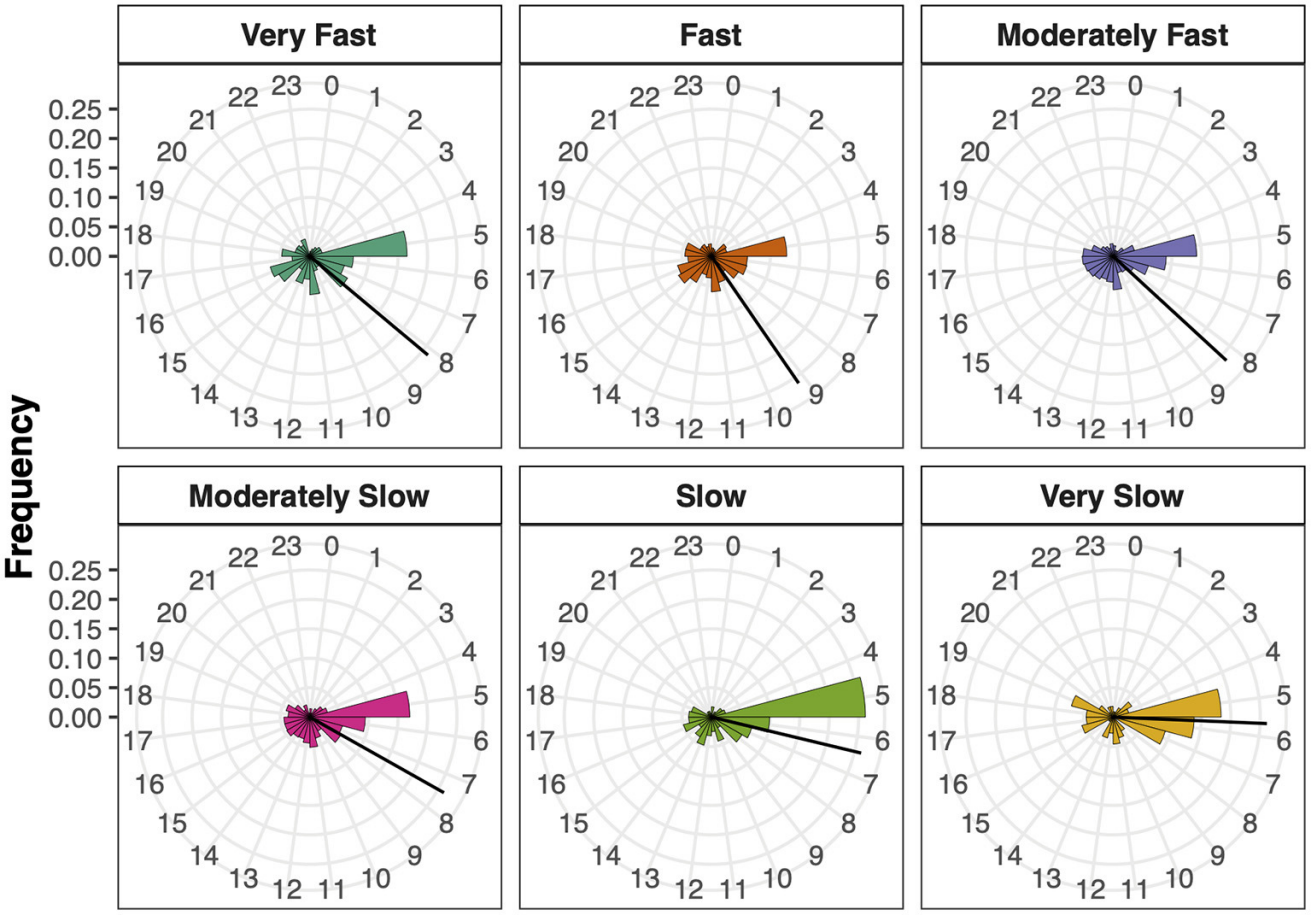
Time of the day per SMT cluster. Bars show the hour of test execution and the bar length shows the frequency per hour. The black lines represent the circular mean per SMT cluster.

In order to test if the Time of the Day differed between the SMT clusters, the hour of test execution from each participant was converted into circular vectors before applying a circular ANOVA. Time of the Day resulted in a significant main effect, *F*_(5, 3, 570)_ = 13.22, *p* < 0.001, η^2^ = 0.20, suggesting that the mean hour of test execution differed between the SMT clusters (Figure 3). *Post-hoc* comparisons (adjusted α = 0.004) resulted in significant differences between the Fast and Moderately Slow (*p* = 0.004), the Fast and Slow (*p* < 0.001) and the Moderately Fast and Slow (*p* < 0.001) SMT clusters. All other comparisons were non-significant (all *p* > 0.004). These differences suggest that the earlier it was during the day, the higher was the probability to observe a slower SMT cluster.

#### Arousal

Arousal did not differ between the SMT clusters, *F*_(5, 3, 570)_ = 1.89, *p* = 0.09, η^2^ = 0.003, suggesting that participants arousal level did not influence the SMT in terms of the cluster ranges.

#### Long-Term Stress

Participants' PSS score did not show a main effect, *F*_(5, 3, 570)_ = 1.18, *p* = 0.32, η^2^ = 0.002, suggesting that long-term stress did not affect the SMT in terms of the cluster ranges.

#### Musical Experience

**Figure 4:**
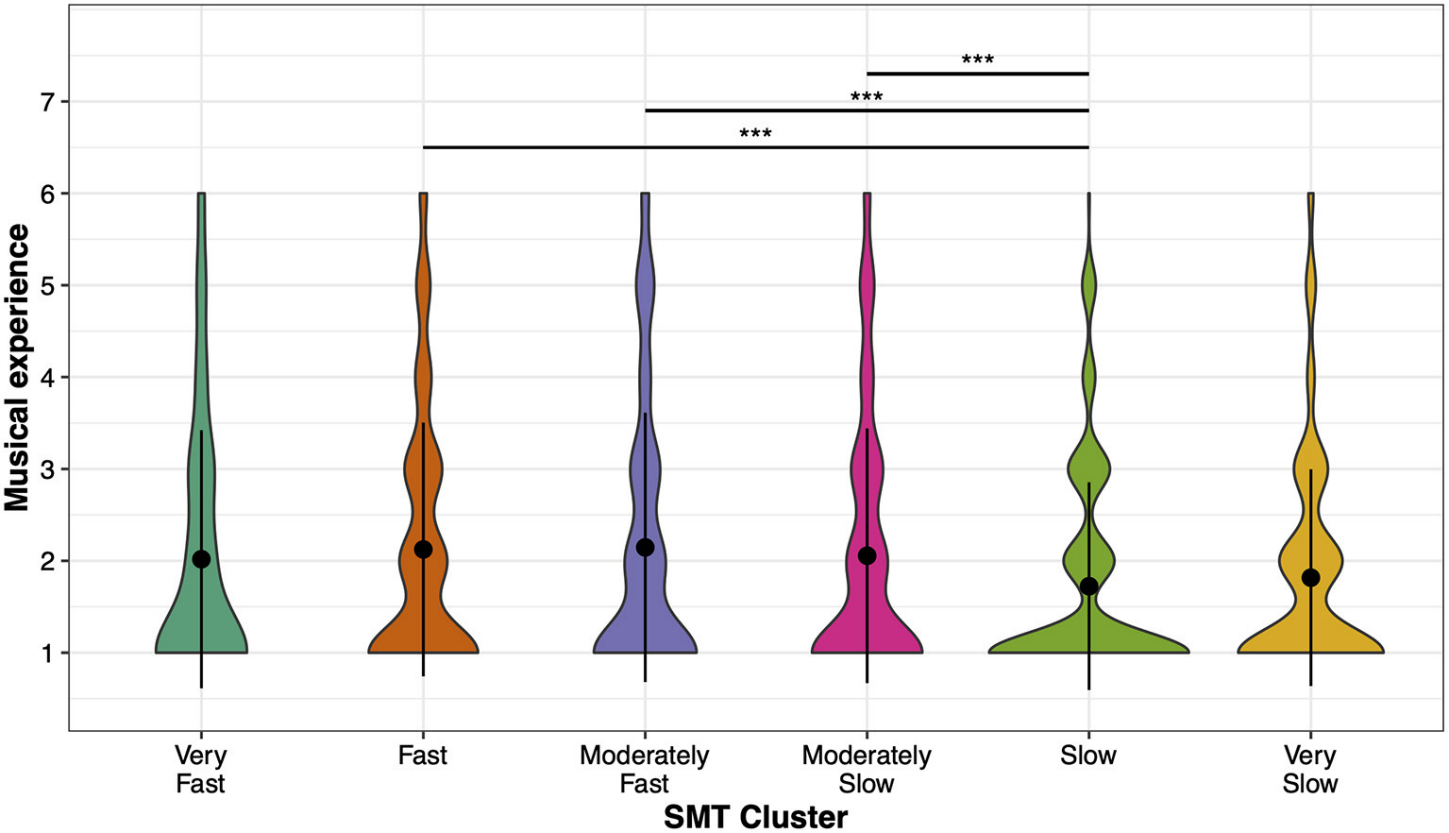
Distribution of musical experience per SMT cluster. Mean values (black points) and standard deviations (error bars) of participants' musical experience for each SMT cluster. Colored areas show the distribution of musical experience. Asterisks indicate significant differences: ***p < 0.001.

The ANOVA on Musical Experience and SMT clusters showed a main effect, *F*_(5, 3, 570)_ = 4.91, *p* < 0.001, η^2^ = 0.007 (Figure 4). *Post-hoc* comparisons show that participants in the Slow SMT cluster had the lowest musical experience, compared to the Fast (*p* < 0.001), Moderately Fast (*p* < 0.001), and Moderately Slow SMT clusters (*p* = 0.003). All other comparisons were non-significant (all *p* > 0.05).

### General Predictors of SMT

In order to predict general differences in SMT, participants' ITIs were cluster-wise z-transformed. Transforming the SMT values per cluster allows for the assessment and comparison of differences for faster or slower participants within each cluster, independently from their overall belonging to a given SMT cluster (i.e., mode). This approach accounted for the different time scales in the clusters. A multiple linear regression was applied in order to predict the z-scored SMT based on age, arousal, long-term stress, and musical experience. The regression was significant, *F*_(4, 3, 570)_ = 3.72, *p* = 0.01, *R*^2^ = 0.004, with Arousal (β = −0.05, *p* = 0.001) and Musical Experience (β = 0.03, *p* = 0.01) as significant predictors. As the estimates show, more aroused participants showed a faster SMT than less aroused participants, and more musically experienced participants showed a slower SMT than less experienced participants across the sample.

## Discussion

With a large-scale online experiment, this study investigated factors influencing the spontaneous motor tempo (SMT), measured as the mean inter-tap interval (ITI), by implementing a finger-tapping paradigm in a web application. Participants were grouped into six different SMT clusters, differing in terms of their mean ITIs, ranging from Very Fast (*M* = 265 ms, *SD* = 74) to Very Slow (*M* = 1,757 ms, *SD* = 166). Results show that the SMT clusters differed by age, suggesting that older participants preferred a slower SMT. The average time of the day of test execution differed between the SMT clusters as well, suggesting that the earlier it was during the day, the slower were participants' SMT. Arousal and long-term stress did not differ between the SMT clusters, yet more aroused participants showed a faster SMT within their SMT clusters. Furthermore, musical experience showed contrasting results when comparing differences between the SMT clusters and prediction across the whole sample. These findings suggests that individuals' SMT depends on age, the time of the day, and arousal.

The first hypothesis stated that SMT slows down with higher age, which was confirmed by our results, as the mean age of participants increased between the Fast (*M* = 525 ms, *SD* = 70) and the Slow (*M* = 1,314 ms, *SD* = 106) SMT clusters. This result confirms previous studies comparing the SMT of different age groups, which also found a slowed down SMT (Provasi and Bobin-Bègue, 2003; Baudouin et al., 2004; McAuley et al., 2006; Monier and Droit-Volet, 2018, 2019). In the current study, participants' age ranged from 7 to 49 years (after the removal of outliers). Whereas it was previously reported that elderly (age 66–94 years) showed SMTs of up to 1,125 ms (Baudouin et al., 2004), our results suggest that such a slowing pace preference exists for younger adults as well. This indicates that the slowing of SMT may already be present in younger to middle-aged adults (age 18–49 years). While the slowing of the intrinsic timekeeper, and in turn an age-related decline in behavioral and processing speed can be assumed for the elderly (Surwillo, 1968), such a decline may start at an earlier age as suggested by this study with a younger sample (age 7–49 years). This is in line with previous studies showing cognitive aging effects for memory and mental speed starting from 20 years on (Salthouse, 2010). Our results might point toward a higher resource demand for fast information processing present in middle-aged adults (up to 49 years), since processing speed is a mediator for working memory and SMT (Baudouin et al., 2004). Future studies could further investigate this by explicitly focusing on the young and middle-aged adults between 20 and 60 years.

The second hypothesis stated that chronobiology, i.e., the time of the day when responding, would influence the pace of the SMT. Previous studies showed a fluctuation of the SMT during the course of a day for finger tapping as well as smartphone tapping speed (Moussay et al., 2002; Huber and Ghosh, 2021). In order to further investigate this effect, we applied circular statistics on the mean ITIs and compared the SMT clusters for the average time of test execution. As the results show, the mean SMT was slower the earlier it was during the day, and the SMT clusters differed from each other. This is in line with a previously reported result suggesting a speeding-up of the SMT from 06:00 in the morning to 18:00 (Moussay et al., 2002). The reason for this fluctuation of the SMT might be the circadian rhythm (i.e., day-night cycle). The biological clock has been shown to influence cognitive and physiological functions such as motor processes, reaction time, time judgements, and memory tasks (Valdez and Ramírez, 2012). It should be noted that in our case, each SMT cluster showed a relatively early average time of the day, which is probably caused by the online implementation of this experiment. As the study gained more visibility (especially in China), a large number of participants took part in a short amount of time, which in our case was in the respective local morning hours. Further research is needed to confirm the SMT fluctuation and its dependence on the circadian rhythm, controlling for the time of the day when assessing the SMT. Furthermore, the influence of the circadian rhythm indicates that the pace of the SMT might depend on the chronotype as well, that is a person's natural inclination for the sleep period of the day, which has been shown to also influence the motor timing of musicians (van Vugt et al., 2013). Our findings suggest that the SMT as measured with a finger-tapping paradigm might be a useful method to assess the circadian rhythm in cognitive performance capabilities.

The third hypothesis stated that arousal and long-term stress would influence the SMT, as a higher arousal level and more long-term stress caused the SMT to speed up. Previous studies showed that induced arousal and physical activity, which in turn leads to a higher arousal level, sped up the pace of SMT (Boltz, 1994; Perilli, 1995; Dosseville et al., 2002). The results of the current study partly confirm this, as the regression model with cluster-wise z-transformed ITIs did suggest a faster SMT with higher arousal across the SMT clusters, yet the arousal level of participants did not differ between the SMT clusters. The reason for this relatively small influence of arousal on the SMT might be due to the nature of this experimental setting. As participants were able to do the test online, it is quite likely that the majority of them were in relatively relaxed situations, for example in front of their desk at home. This assumption is further supported by the relatively low average arousal level. As long-term or chronic stress leads to general physiological changes such as a higher heart rate over longer time periods (Yaribeygi et al., 2017), we assumed a generally higher arousal level in participants with more long-term stress, and in turn a faster SMT. We could not find any influence of long-term stress, assessed by the 4-item *Perceived stress scale* (PSS), as neither the SMT clusters differed from each other nor did the PSS score show an effect in the regression model. This suggests that the SMT reflects short-term stress or physiological states and is not affected by long-term stress conditions. An explanation might be that physiological arousal is caused by adrenaline (and other hormones) that directly affect the neural circuits involved in the intrinsic timekeeper, and also leads to increased pulse and blood pressure, thus they are epiphenomena of arousal. Long-term stress does not necessarily result in momentary stress and in turn higher physiological arousal. The effects of long-term or chronic stress on heart rate are due to long-term adaptions to higher average adrenalin levels (note that we were using self-report measures of arousal and long-term stress, not physiological ones). Future studies might investigate the effect of arousal in a way which clearly differentiates between physiological and perceived as well as short- and long-term arousal states as previous studies' results are inconclusive (Dosseville et al., 2002; Sysoeva et al., 2013).

Previous studies reported an effect of musical experience on the SMT as children with musical experience showed slower SMTs than children without musical experience, yet no differences were found between adult musicians and non-musicians (Drake et al., 2000). This finding together with a slower production rate of melodies for musicians have been linked to the perceptual capability to organize events into longer time spans, which is reflected in a greater accuracy and rate range for sensorimotor synchronization to musical rhythms (Repp and Doggett, 2007; Repp, 2010; Martens, 2011; Scheurich et al., 2018; Hammerschmidt and Wöllner, 2020). Our results did not confirm the assumption of a slower SMT for musically experienced individuals, since the least experienced participants were in the Slow SMT cluster. On the contrary, the regression model of cluster-wise z-transformed SMTs suggested that musically more experienced participants in each SMT cluster preferred a slower SMT. Thus, a potential influence of musical experience on the SMT, its direction and underlying cause warrants further and more detailed investigations.

Compared to previous studies reporting SMTs ranging from 190 to over 950 ms for healthy adults (Fraisse, 1982; Collyer et al., 1994; Moelants, 2002), SMT variability was much larger in our study (range: 123–2,150 ms). A possible explanation for the very long ITIs might be that some of the participants did not follow the task instruction correctly and mentally subdivided their finger taps, for example, only carrying out every other tap physically. Furthermore, the distribution of ITIs was multimodal, showing clusters for mostly slower SMTs as well, drawing a more complex picture of the SMT than previously reported. This is an interesting result in itself, as the mean periods of SMT clusters could be approximate multiples of about 250 ms (mean absolute deviation from the closest value nT with *n* ϵ {1, 2, 3, 4, 5, 7} and *T* = 251 ms was 17 ms, range 0–59 ms). This result might be indicative for an internal oscillator with a base frequency around 4 Hz (van Noorden and Moelants, 1999; Ding et al., 2017). Thus, lower modes (subharmonics) of this base frequency might have been used for the finger-tapping task (every second, third, fourth, etc. oscillatory peak). The SMT cluster with *T* ~ 500 ms (second mode) seems to be most common for the SMT as previous studies found a clustering around 500–600 ms, which corresponds with the Fast SMT cluster in our study, ranging from 375 to 642 ms (*M* = 525 ms, *SD* = 70) with the highest number of participants. The clustering around this potential second mode might be due to anatomical and biomechanical properties of the body (Goodman et al., 2000; Todd et al., 2007). This also seems be in line with the resonance frequency model of pulse perception (van Noorden and Moelants, 1999). The small effect sizes for the factors age and musical experience might further indicate that the modulation of the SMT is relatively small and the preference for a certain pace of the SMT is, indeed, quite stable.

The larger variability of SMT and the possible explanation of mental subdivision of finger tapping points to a limitation of this study: Due to the online implementation it was not possible to observe and control for task comprehension and task execution. For example, participants in the Very Fast SMT cluster could have used two fingers and or deliberately did not follow task instructions. On the other hand, the online implementation resulted in a large number of participants, keeping the potential influence and likelihood of false responses relatively small, and outliers had been excluded before analyses. Compared to other studies, a relatively short recording time of 15 seconds of the SMT was chosen in order to reduce dropout rate by keeping motivation in the study high. In order to account for this, a relatively strict cut-off value for tapping variability was implemented, thus ensuring that participants kept their tapping pace constant. The relatively simple measures for musical experience and arousal do not provide much detail in the respective domains, yet they were chosen to keep the experiment length relatively short in order to reduce the dropout rate of the experiment (Hoerger, 2010), as they are easy to understand for people not typically participating in scientific studies and coming from different backgrounds. Furthermore, it was not possible to control the distribution of participants based on specific demographics. The experiment was relatively short (3–5 min) and 67% completed the whole experiment. The dropout rate might have been further reduced when the feedback of tapping performance would have been given after participants were asked to rate their arousal level, musical experience, and the long-term stress (part 3 of the experiment).

To conclude, this study investigated factors influencing the spontaneous motor tempo (SMT) in a large-scale online experiment by implementing the finger-tapping paradigm in a self-developed web application. Results confirmed a slowing with age effect on the SMT and showed an influence of the time of the day, indicating that the earlier it is during the day, the slower is the SMT. This suggest that the SMT might be a useful method for the assessment of someone's circadian rhythm. Arousal only showed a small effect on the SMT, which might be due to the test conditions, as participants might have done the test in relatively relaxed situations. Whereas all these effects have a physiological basis, musical experience showed a more complex influence on the SMT than previous studies have suggested, which warrants further investigations. Thus, this study's methodological approach and outcomes are informative for the psychology of time and music, showing a complex relationship of factors and their effect on multimodal SMT distribution with a large number of individuals.

## Data Availability Statement

The raw data supporting the conclusions of this article are openly available in Zenodo repository at http://doi.org/10.5281/zenodo.4897921.

## Ethics Statement

The studies involving human participants were reviewed and approved by Ethics Committee of the Faculty of Humanities at University of Hamburg. Written informed consent from the participants' legal guardian/next of kin was not required to participate in this study in accordance with the national legislation and the institutional requirements.

## Author Contributions

DH and CW designed the research. DH and KF analyzed the data. DH wrote most parts of the manuscript. All authors reviewed the manuscript.

## Conflict of Interest

The authors declare that the research was conducted in the absence of any commercial or financial relationships that could be construed as a potential conflict of interest.
