# Supplementary material for: Spontaneous Motor Tempo: Investigating Psychological, Chronobiological, and Demographic Factors in a Large-Scale Online Tapping Experiment
Source: Front Psychol. 2021 Jun 22;12:677201. doi: 10.3389/fpsyg.2021.677201 (PMC8262453; doi:10.3389/fpsyg.2021.677201)
Supplement: Supplementary file 1 [file Data_Sheet_1.pdf]

Supplementary Material

1 Experimental Questions

Part 1 (Demographics)

1. Which year were you born? (text box)

2. What is your gender? (dropdown)
   - male
   - female
   - other

3. Did you partake in this study before? (dropdown)
   - yes
   - no

4. In which country have you spent most of your life? (dropdown with list of countries)

5. How large is the population of the area you currently live in? (dropdown)
   - more than 10 million people
   - 3 to 10 million people
   - 1 to 3 million people
   - 300,000 to 1 million people
   - 100,000 to 300,000 people
   - 20,000 to 100,000 people
   - 1,000 to 20,000 people
   - 100 to 1,000 people
   - less than 100 people

Part 2 (Tapping task)

Instructions:
Your task is now to tap steadily for 15 seconds. Try to keep the time between each tap as even as possible. Choose a pace that feels most comfortable and natural to you right now.

When using a touchscreen display, please use your finger to tap in the circle that will appear below. If you use a computer, you can use any key on your keyboard or use the mouse to click into the circle. Please click START when you are ready. The experiment timer will then start running with your first tap.

Feedback for unsuccessful trail:

Sorry, but your taps were either not even enough to too few. Remember, try to pace taps evenly at your most comfortable rate for as long as indicated by the timer.

- Number of taps: XX (min. 8 taps required)
- Evenness of taps: XX% (min. 90.2% required)

You need to tap as long as the timer is running, please try again.

Feedback for successful trial:

Very good! Your taps were even, please click CONTINUE, we have a few more questions for you.

- Number of taps: XX
- Evenness of taps: XX%

Part 3 (Musical experience, arousal, work load, PSS-4)

6. Do you make music? (check box)
   - never
   - almost never
   - sometimes
   - fairly often
   - very often
   - I am a professional

7. How do you feel right now? (check box)
   - very calm
   - rather calm
   - neutral
   - rather excited
   - very excited

8. How many hours do you spend studying/working in a typical week? (dropdown)
   - none
   - up to 10 hours
   - up to 20 hours
   - up to 30 hours
   - up to 40 hours
   - up to 50 hours
   - more than 50 hours

   The following questions are about your feelings and thoughts during the last month. You will be asked to indicate how often you felt or thought a certain way. They may explain
   your inner timing.

9. In the last month, how often have you felt that you were unable to control the important things in your life? (check box)
   - never
   - almost never
   - sometimes
   - fairly often
   - very often

10. In the last month, how often have you felt confident about your ability to handle your personal problems? (check box)
   - never
   - almost never
   - sometimes
   - fairly often
   - very often

11. In the last month, how often have you felt that things were going your way? (check box)
   - never
   - almost never
   - sometimes
   - fairly often
   - very often

12. In the last month, how often have you felt difficulties were piling up so high that you could not overcome them? (check box)
   - never
   - almost never
   - sometimes
   - fairly often
   - very often

2 Graphical User Interface

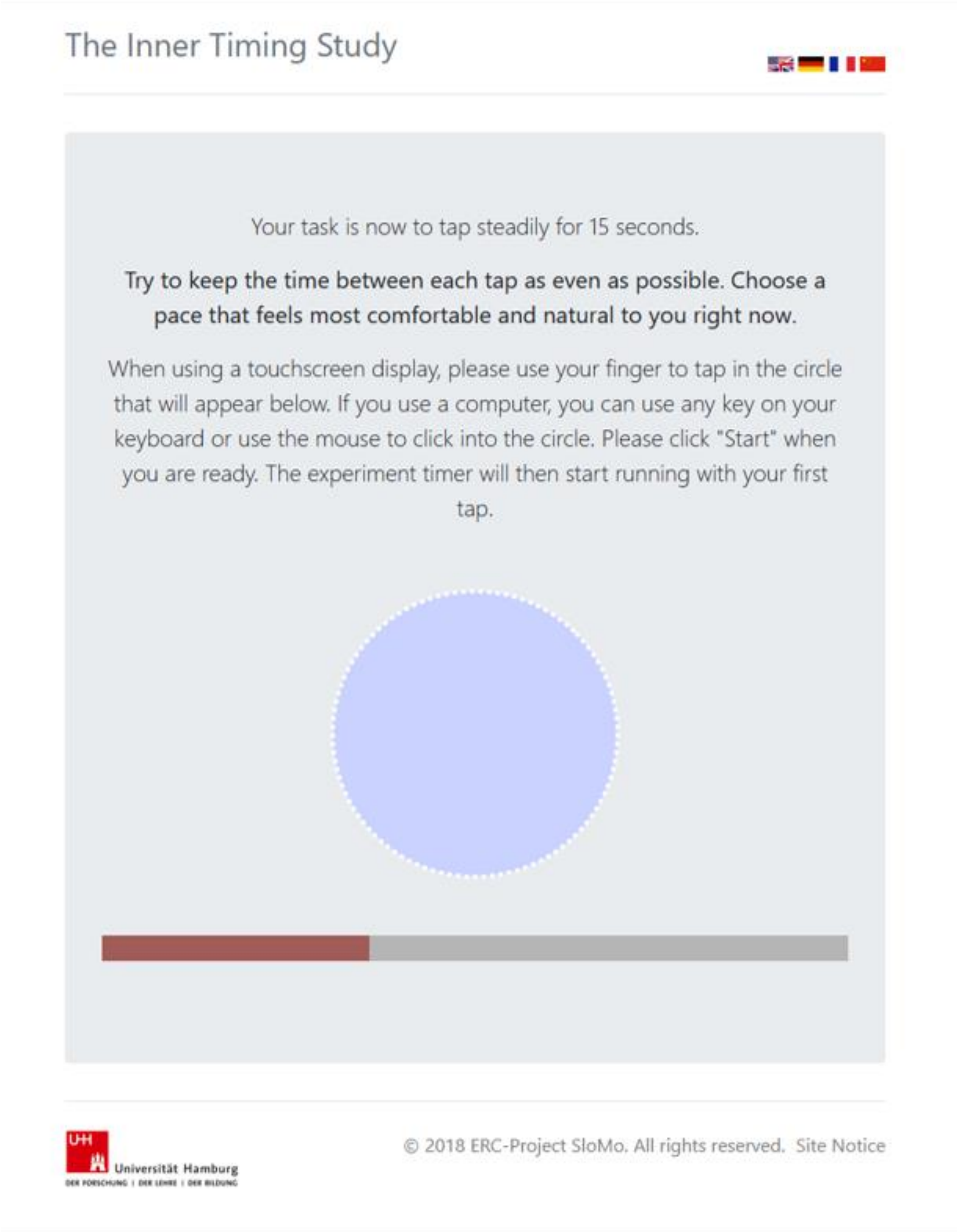

Supplementary Figure 1. Graphical user interface of the finger-tapping task. This figure shows the task description and the circle participants tapped on when using a touchscreen device or computer mouse. After a tap, the circle flashed shortly for visual feedback which was also the case when a keyboard or mouse was used. The horizontal bar at the bottom was the timer indicating for how much longer participants needed to tap (red bar moving from left to right).

3 Distribution of Device and Software Types

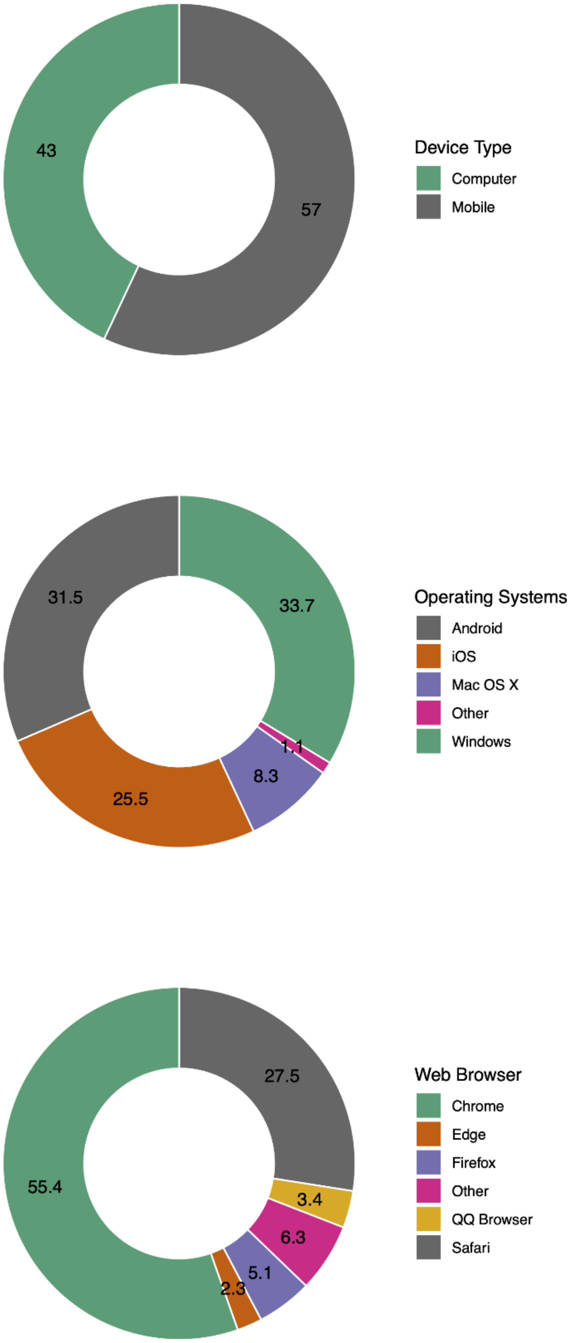

Supplementary Figure 2. Distribution of device type (top circle), operating systems (middle circle), and web browser (bottom circle) used by participants. The values inside the circle indicate percentages based on $N = 3,576$. 'User agents' headers were parsed using the uaparserjs package in R.
